# Neurologic Abnormalities in Mouse Models of the Lysosomal Storage Disorders Mucolipidosis II and Mucolipidosis III γ

**DOI:** 10.1371/journal.pone.0109768

**Published:** 2014-10-14

**Authors:** Rachel A. Idol, David F. Wozniak, Hideji Fujiwara, Carla M. Yuede, Daniel S. Ory, Stuart Kornfeld, Peter Vogel

**Affiliations:** 1 Department of Internal Medicine, Washington University School of Medicine, St. Louis, Missouri, United States of America; 2 Department of Psychiatry, Washington University School of Medicine, St. Louis, Missouri, United States of America; 3 Diabetic Cardiovascular Disease Center, Washington University School of Medicine, St. Louis, Missouri, United States of America; 4 Department of Neurology, Washington University School of Medicine, St. Louis, Missouri, United States of America; 5 Department of Pathology, St. Jude Children's Research Hospital, Memphis, Tennessee, United States of America; University of Cincinnati, United States of America

## Abstract

UDP-GlcNAc:lysosomal enzyme *N*-acetylglucosamine-1-phosphotransferase is an α_2_β_2_γ_2_ hexameric enzyme that catalyzes the synthesis of the mannose 6-phosphate targeting signal on lysosomal hydrolases. Mutations in the α/β subunit precursor gene cause the severe lysosomal storage disorder mucolipidosis II (ML II) or the more moderate mucolipidosis III alpha/beta (ML III α/β), while mutations in the γ subunit gene cause the mildest disorder, mucolipidosis III gamma (ML III γ). Here we report neurologic consequences of mouse models of ML II and ML III γ. The ML II mice have a total loss of acid hydrolase phosphorylation, which results in depletion of acid hydrolases in mesenchymal-derived cells. The ML III γ mice retain partial phosphorylation. However, in both cases, total brain extracts have normal or near normal activity of many acid hydrolases reflecting mannose 6-phosphate-independent lysosomal targeting pathways. While behavioral deficits occur in both models, the onset of these changes occurs sooner and the severity is greater in the ML II mice. The ML II mice undergo progressive neurodegeneration with neuronal loss, astrocytosis, microgliosis and Purkinje cell depletion which was evident at 4 months whereas ML III γ mice have only mild to moderate astrocytosis and microgliosis at 12 months. Both models accumulate the ganglioside GM2, but only ML II mice accumulate fucosylated glycans. We conclude that in spite of active mannose 6-phosphate-independent targeting pathways in the brain, there are cell types that require at least partial phosphorylation function to avoid lysosomal dysfunction and the associated neurodegeneration and behavioral impairments.

## Introduction

Mucolipidosis II (ML II) and mucolipidosis III (ML III) are autosomal recessive lysosomal storage diseases caused by a deficiency of UDP-GlcNAc:lysosomal enzyme *N*-acetylglucosamine-1-phosphotransferase (GlcNAc-1-phosphotransferase), an α_2_β_2_γ_2_ hexameric enzyme that catalyzes the first step in the synthesis of the mannose 6-phosphate (Man-6-P) targeting signal on lysosomal hydrolases which is required for their targeting to lysosomes in many cell types [1]. GlcNAc-1-phosphotransferase is encoded by two genes, *GNPTAB* and *GNPTG*, which give rise to the α/β subunit precursor and the γ subunit, respectively. The α/β subunits contain the catalytic function of the enzyme, whereas the γ subunit enhances the phosphorylation of a subset of lysosomal hydrolases [2]. In humans, mutations in *GNPTAB* give rise to ML II (MIM# 252500) or ML III α/β (MIM# 252600), whereas mutations in *GNPTG* cause ML III γ (MIM# 252605) [3]. Patients with the rapidly progressive and severe ML II exhibit a total or near total loss of GlcNAc-1-phosphotransferase activity mostly due to nonsense or frameshift mutations while ML III α/β patients tend to have missense or splice-site mutations resulting in some residual GlcNAc-1-phosphotransferase activity and a milder clinical course [4]. Patients with ML III γ retain the most phosphorylation activity of the three disorders and have the mildest course. In fibroblasts and other mesenchymal cells of the patients, lysosomal acid hydrolases which lack the Man-6-P recognition marker are not properly sorted to the lysosome but rather secreted into the patient's blood stream. The resultant low intracellular levels of these acid hydrolases lead to lysosomal dysfunction and accumulation of undegraded material. However, it has been shown that some non-mesenchymal cells have the ability to traffic lysosomal hydrolases to lysosomes via Man-6-P-independent mechanisms [5]. Thus, the level of some lysosomal hydrolases has been reported to be normal or near normal in total brain, liver and muscle of ML II patients [6], [7]. In spite of this, these patients exhibit substantial neurologic manifestations including early cessation of neuromotor development, limited verbal expression, and moderate intellectual disability [4]. To better understand the consequences of complete and partial loss of the Man-6-P-targeting signal on neurologic function and brain pathology, we performed neurological characterization of ML II *(Gnptab^−/−^)* and ML III γ *(Gnptg^−/−^)* mouse models [8], [9]. ML II mice have a total loss of acid hydrolase phosphorylation whereas ML III γ mice have a variable decrease in hydrolase phosphorylation [2], [10]. Nevertheless, in both cases, the activity of multiple acid hydrolases in brain extracts are normal or only moderately decreased [10], [11], as previously reported for human patients [6], [7]. Here we report the results of these studies and compare the neurologic abnormalities we found with those reported by Kollmann *et al.* [12] in a different mouse model of ML II.

## Materials and Methods

### Mice

All mice were housed in a barrier facility at 24°C on a fixed 12-hour light and 12-hour dark cycle and were fed rodent chow #5001 (Purina, St. Louis, MO) ad libitum. All protocols involving the use of animals were in compliance with the National Institutes of Health's Guide for the Care and Use of Laboratory Animals and approved by the Animal Studies Committee in the Division of Comparative Medicine at Washington University School of Medicine in St. Louis (Protocol #20130010). Mice were housed in a barrier facility maintained under standards meeting federal, state and local guidelines and under the supervision of licensed veterinarians. We have previously reported the generation and initial characterization of the *Gnptab^−/−^* [8] and *Gnptg^−/−^* mice [10]. The *Gnptab^−/−^* and *Gnptg^−/−^* mice are maintained in a C57Bl/6-129S5/SvEvBrd background. Mice were genotyped as previously described to identify WT or knockout mice for use in this study [8], [10].

### Behavioral Testing

Based on preliminary behavioral observations, *Gnptab^−/−^* and *Gnptg^−/−^* mice were evaluated on a 1h locomotor activity test, a battery of sensorimotor measures and the rotarod test (in that order) to assess possible age-related impairments in motor/sensorimotor functions. In the cohort of *Gnptab^−/−^* mice and their WT littermate controls, the sensorimotor battery was conducted 1 day after the activity test with the rotarod test being conducted 1 day after the sensorimotor battery when the mice were evaluated at 1 month of age. The same was true for the testing conducted at 4–5 months of age except that the 1h activity test was not conducted at this age. The same sequence of tests was used for the cohort of *Gnptg^−/−^* and their strain and age-matched WT control mice although the times intervening between tests were different as were the ages at testing (4–6 and 12–14 months). In this latter cohort, the sensorimotor battery was conducted within 48 h of the activity test and the rotarod test was conducted 12–14 days after the activity test.

### 1h locomotor activity and sensorimotor battery

Locomotor activity was evaluated in the mice using transparent (47.6 × 25.4 × 20.6 Cm high) polystyrene enclosures and computerized photobeam instrumentation as previously described [13]]. General activity variables (total ambulations, vertical rearings) along with indices of emotionality including time spent, distance traveled and entries made in a 33 × 11 cm central zone were analyzed. All mice were also evaluated on a battery of sensorimotor tests designed to assess balance (ledge and platform), strength (inverted screen), coordination (pole and inclined screens) and initiation of movement (walking initiation), as previously described [13], [14].

### Rotarod

Motor coordination and balance were further studied using procedures similar to our previously published methods [14], [15]. Our rotarod protocol included three conditions: a stationary rod; a rod that rotated at a constant speed (2.5 rpm for 60 s maximum); and a rod that rotated at an accelerating speed (2.5 – 10.5 rpm over 1–180 s maximum). The protocol consisted of three training sessions with each session including one stationary rod trial, two constant speed rotarod trials, and two accelerating speed rotarod trials. Sessions were separated by 4 days to minimize motor learning and time spent on the rod was used as the dependent variable to assess performance. Note that one mouse from the *Gnptab^−/−^* group was deleted from the data set because it appeared to have difficulty moving one of its forepaws and another mouse was deleted from the WT control group because it climbed over different parts of the apparatus making it impossible to record valid times on the rod.

### Statistical analyses for behavioral data

Analysis of variance (ANOVA) models were used to analyze the behavioral data. Repeated measures (rm) ANOVA models containing two between-subjects variables (Genotype and Sex) and one within-subjects (repeated measures) variable (i.e., Time Blocks) were used to analyze the ambulatory activity and vertical rearing data. A similar model was used to analyze the rotarod data although two within-subjects variables (i.e., trials and sessions) were included to analyze the constant speed and accelerating rotarod data. Typically, one-way ANOVA models were used to analyze differences between groups for the other activity variables and measures in the sensorimotor battery. The Huynh-Feldt adjustment of α levels was utilized for all within-subjects effects containing more than two levels to protect against violations of sphericity/compound symmetry assumptions underlying rmANOVA models. Pairwise comparisons were conducted following relevant, significant overall ANOVA effects, which were subjected to Bonferroni correction when appropriate, although planned comparisons were also conducted.

### Immunohistochemistry

Mice of the indicated ages were anesthetized and perfused through the heart with 10% neutral buffered formalin (Sigma, St. Louis, MO) and fixed for 4 days in 10% neutral buffered formalin (Sigma, St. Louis, MO) at RT. The brain and spinal cords of perfused mice were left in place within the skull and vertebral columns, respectively, and post-fixed by immersion in 10% buffered Formalin for at least 24 h before being decalcified in formic acid (TBD-2 Decalcifier; ThermoFisher Scientific, Waltham, MA). Coronal sections of brain and spinal cord were embedded in paraffin, sectioned at 4 µm, mounted on positively charged glass slides (Superfrost Plus; Thermo Fisher Scientific, Waltham, MA), and dried in a 60°C oven for 20 min. Antigen retrieval on tissue sections was completed in a pressure cooker (Decloaking Chamber; Biocare Medical, Concord, CA) in citrate buffer, pH 6 for 15 min at 110°C, followed by incubation with the primary antibodies. The primary antibodies used in this study included: anti-Iba1 (ionizing calcium-binding adaptor molecule 1: CP290A; BioCare Medical) diluted to 1∶250 and applied for 2 h at RT; anti-GFAP *(glial fibrillary acidic protein: Z0334*; Dako, Carpinteria, CA), diluted to 1∶1500 and applied for 1 h at RT; anti-LAMP-1 (lysosomal-associated membrane protein 1 ab24170; Abcam, Cambridge, MA), diluted 1∶400 and applied for 1 h at RT; anti-LC3B (microtubule-associated protein 1 light chain-3B: I7543, Sigma, St. Louis, MO), diluted 1∶2000 and applied for 2 h at RT; anti-MAC2 (macrophage cell-surface protein 2: ACL-8942AP, Accurate, Westbury, NY) diluted 1∶20,000 and applied for 30 min at RT; anti-NFP (M0762; Dako) diluted 1∶40 and applied for 2 hours at RT; and anti-ubiquitin (Z0458, Dako) diluted 1∶800 and applied for 15 min at RT. Binding of primary antibodies (GFAP, IBA1, LAMP-1, LC3B) was visualized using rabbit or rodent polymeric horseradish peroxidase method (BioCare Medical, Concord, CA) on a LabVision stainer (Thermo Fisher Scientific, Freemont, CA). Binding of MAC2 and NFP was visualized using streptavidin horseradish peroxidase (Thermo Fisher), with 3,3′-diaminobenzidine as the chromogenic substrate and a light hematoxylin counterstain. Binding of primary antibody for ubiquitin was visualized using Bond Polymer Refine Red Detection Kit (Leica, Bannockburn, IL).

### Lipid Analysis

Mice of ages indicated were anesthetized and perfused through the heart with PBS, brain tissues isolated and frozen at -80°C until used. Sample preparation and quantification of sphingolipid species by tandem mass spectrometry (LC-MS/MS) was performed as previously described [16].

### Determination of amino sugars content

Mice of ages indicated were anesthetized and perfused through the heart with PBS, brain tissues isolated and frozen at −80°C until used. Whole brains were homogenized in water, sonicated and an aliquot was saved to determine the protein concentration. The remaining lysate was brought to 4N Hcl (Sigma, St. Louis, MO), heated in a sand bath at 100°C for 4 h, lyophilized, re-suspended in 1mL water, and insoluble material spun down at 25,000X*g* for 10 min. The supernatant was isolated and stored at −20°C. Amino sugar content was determined by the Reissig assay [17] with known concentrations of *N*-Acetylglucosamine (Sigma, St. Louis, MO) used as a standard. Each sample was normalized to protein concentration and data plotted as nmol amino sugars/mg total protein.

### Determination of fucose content

Mice of ages indicated were anesthetized and perfused through the heart with PBS, brain tissues isolated and frozen at −80°C until used. Whole brains were homogenized in water, sonicated and an aliquot saved for protein concentration. The remaining lysate was adjusted to pH 1.0 with 1M H_2_SO_4_ (Sigma, St. Louis, MO), heated in a sand bath at 100°C for two h, neutralized with barium hydroxide (Sigma, St. Louis, MO), and the volume adjusted to 1 mL with water. After 30 min at 4°C, samples were spun down at 25,000X*g* for 10 min and the supernatant isolated and stored at −20°C. Fucose content was determined by the Dische assay [18] with known concentrations of fucose (Sigma, St. Louis, MO) used as a standard. Each sample was normalized to protein concentration and the data plotted as nmol fucose/mg total protein.

### Fucosidase activity assay

Mice were anesthetized and perfused through the heart with PBS, brain tissues isolated and frozen at −80°C until used. Whole brains were homogenized in PBS containing 1% Triton-X (Sigma, St. Louis, MO) and protease inhibitors (complete mini protease inhibitor tablets; Roche, Penzberg, Germany), placed on ice for 15 min, sonicated and centrifuged for 10 min at 25,000X*g*. An aliquot of the supernatant was reserved for protein determination and fucosidase activity of the supernatant was determined by fluorometic enzyme assay as previously described [10]. Activity was expressed as nmoles of hydrolyzed methylumbelliferone per mg protein per h.

## Results

### Behavioral Analyses in *Gnptab^−/−^* and *Gnptg^−/−^* mice

Before starting the behavioral tests to investigate the impact of complete or partial acid hydrolase Man-6-P deficiency on motor and sensorimotor functions, we noted that the *Gnptab^−/−^* mice, but not the *Gnptg^−/−^* mice, developed a hind limb clasping response between 4–6 months of age, suggestive of brain and/or spinal cord abnormalities [19]. In view of this, we initiated the behavioral testing at 1 month of age in the *Gnptab^−/−^* mice to determine whether more subtle motor/sensorimotor impairments were present during the juvenile period long before the clasping response became apparent during early adulthood. The same cohort of *Gnptab^−/−^* mice was retested at 4–5 months to assess the progression of functional impairment. We also focused on tests that did not rely heavily on visual function since the *Gnptab^−/−^* mice have progressive retinal degeneration starting at 2–3 months with complete photoreceptor degeneration by 10 months [8]. The *Gnptg^−/−^* mice were assessed on these same tests as young adults (4–6 months) and in middle age (12–14 months).

### *Gnptab^−/−^* mice exhibit sensorimotor deficits that progress with age

**Figure 1:**
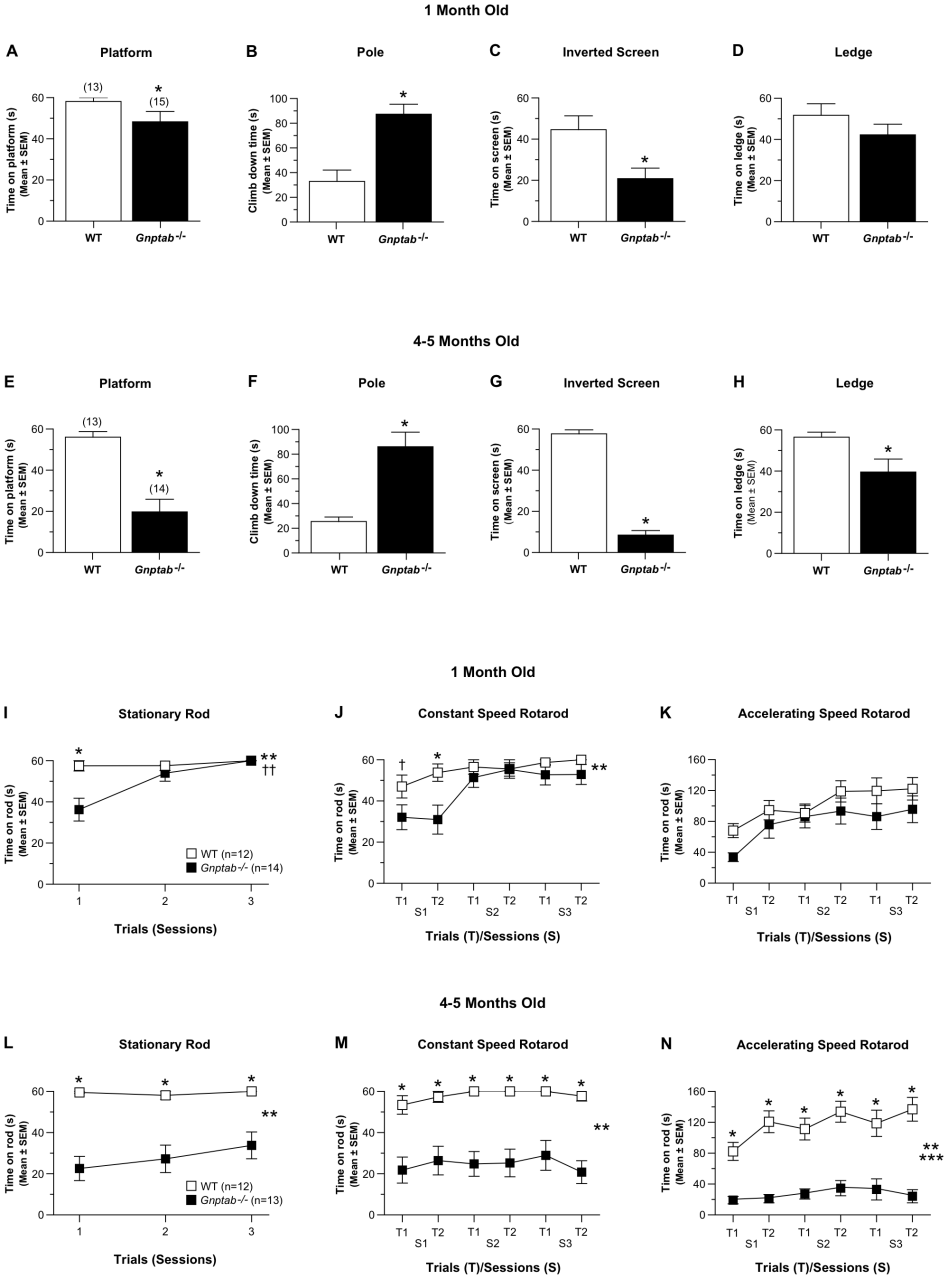
*Gnptab^−/−^* mice exhibit performance deficits on sensorimotor and rotarod tests which show progressive impairment with age. (A–D) At 1-month of age, the *Gnptab^−/−^* mice spent significantly (*p = 0.026) less time on an elevated platform (A), took significantly (*p  =  0.0007) longer to climb down a pole (B), and spent significantly (*p  =  0.020) less time hanging upside down on an inverted screen (C) compared to WT littermate control mice. The *Gnptab^−/−^* mice also showed a nonsignificant trend in terms of spending less time on an elevated ledge (D). (E-H) Larger performance deficits were observed when the mice were tested at 4-5 months of age when significant impairment was observed in the *Gnptab^−/−^* group compared to WT controls on the platform (E; *p  =  0.0002), pole (F; *p  =  0.0005), inverted screen (G; *p <0.00005), and ledge (H; *p  =  0.001) tests (see Fig. 3A for details of sex effects on the ledge test). (I-K) The *Gnptab^−/−^* mice demonstrated mild performance impairments on the rotarod when they were tested at 1-month of age. (I) For example, the *Gnptab^−/−^* mice spent significantly less time on the stationary rod component of the test compared to the WT group (genotype effect, ††*p*  =  0.002); genotype x trials interaction (**p  =  0.0004), but this was mostly due to differences observed on trial 1 (*p  =  0.0007). (J) Analysis of the constant speed rotarod data showed that large differences were observed between the *Gnptab^−/−^* and WT groups but only during the first session (genotype x sessions interaction; **p  =  0.024); trial 1 (†*p*  =  0.046); trial 2 (*p  =  0.022). (K) No significant effects involving Genotype were found as a result of analyzing the accelerating rotarod data. (L-M) The *Gnptab^−/−^* mice showed severely impaired performance on the rotarod tasks when tested at 4–5 months of age. Specifically, the *Gnptab^−/−^* mice were significantly impaired on the stationary rod (L; genotype effect: **p <0.00005; pairwise comparisons for each trial: *p <0.0005), the constant speed rotarod (M; genotype effect: **p <0.00005; pairwise comparisons for each trial: *p <0.003), and accelerating rotarod (N; genotype effect: ***p <0.00005; genotype x trials interaction: **p <0.010; pairwise comparisons for each trial: *p <0.003).

**Figure 2:**
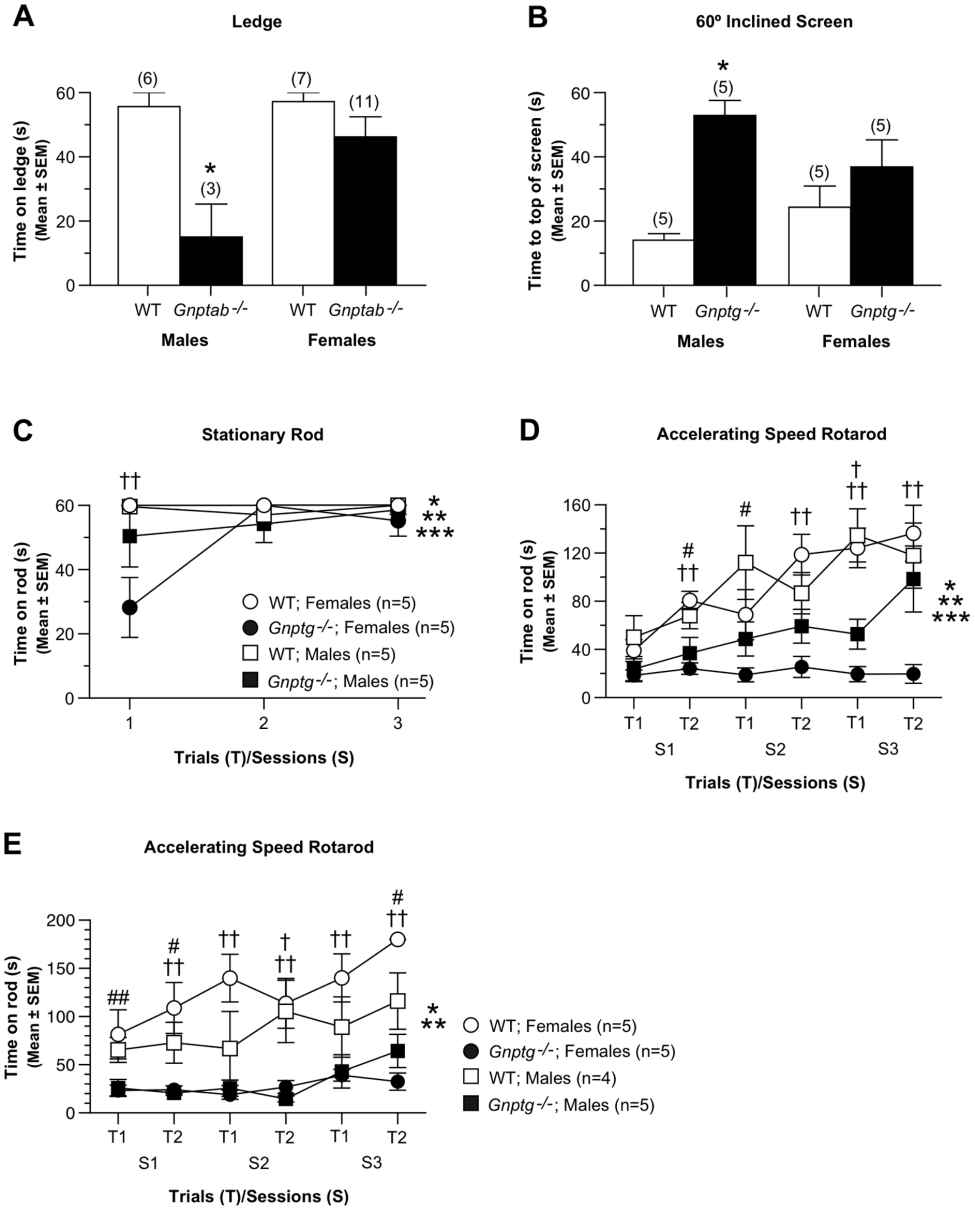
Sex effects on behavior in *Gnptab^−/−^* and *Gnptg^−/−^* mice. (A) In addition to a significant genotype effect, an ANOVA conducted on the ledge data from the *Gnptab^−/−^* and WT control mice at 1 month of age revealed a significant sex effect (*p*  =  0.025) and a genotype x sex interaction (*p*  =  0.041). Subsequent contrasts showed that male *Gnptab^−/−^* mice were significantly impaired on the ledge test compared to the male WT mice [F(1,23)  =  12.98, *p  =  0.002], while differences were not significant between the female groups. (B) Analysis of the 60° inclined screen data from the 4-6 month old *Gnptg^−/−^* mice showed a significant genotype x sex interaction (*p*  =  0.041) in addition to the significant genotype effect, suggesting that impaired performance may have varied differentially as a function of sex in each group. Additional contrasts documented that the male *Gnptg^−/−^* mice took significantly longer to reach the top of the apparatus compared to the males from the WT group [F(1,16)  =  21.51, *p  =  0.0003), while the performance of the females from the two groups did not differ significantly. (C) An rmANOVA on the stationary rod data from testing the *Gnptg^−/−^* and control mice at 4–6 months of age revealed a significant sex x trials interaction, (**p  =  0.030) as well as a significant (*) genotype effect and a significant (***) genotype x trials interaction, suggesting that differences in performance were dependent on trials and sex. Additional contrasts showed that the performance of the male groups of mice did not differ across the stationary rod trials, although the female *Gnptg^−/−^* mice were significantly impaired compared to the WT controls, [F(1,16)  =  6.72, *p*  =  0.020), with significant differences being observed on the first trial (††*p*  =  0.004). (D) Robust performance impairments in the *Gnptg^−/−^* mice at 4-6 months of age were observed during the accelerating rotarod test where an rmANOVA yielded a significant genotype x sex x trials interaction, (***p  =  0.004), along with a significant (*) genotype effect and significant (**) genotype x sessions interaction. Subsequent contrasts relating to the sex effect showed that the male *Gnptg^−/−^* mice remained on the rod longer than the male control group [F(1,16)  =  5.64, *p*  =  0.031) with significant differences occurring on session 3 - trial 1 (†*p*  =  0.002), although large differences were also found for session 1 - trial 2 (#*p*  =  0.036) and session 2 - trial 1 (#*p*  =  0.039). Differences were even greater in the female groups whereby the female *Gnptg^−/−^* mice performed significantly worse than the control females [F(1,16)  =  17.65, *p*  =  0.0007), with significant differences being observed for session 1 - trial 2 (††*p*  =  0.0007), session 2 - trial 2 (††*p*  =  0.0004), session 3 - trial 1 (††*p*  =  0.0002), and session 3 - trial 2 (††*p*  =  0.002). (E) Analysis of the accelerating rotarod data at 12–14 months revealed a significant genotype x gender x trials x sessions interaction, (**p  =  0.017), as well as a significant (*) genotype effect. Additional contrasts related to the sex variable showed that the female *Gnptg^−/−^* mice exhibited significantly inferior performance on average across trials and sessions compared to the female WT group [F(1,15)  =  25.37, *p*  =  0.0001). Pair-wise comparisons revealed significant differences between the female groups on every trial (††*p* <0.007) except session 1 - trial 1 (##*p*  =  0.016), where differences were also very large. The performance of the male *Gnptg^−/−^* mice was also significantly compromised relative to the male WT controls on average across trials and sessions, although the differences were smaller [F(1,15)  =  6.48, *p*  =  0.022). Pair-wise comparisons showed that the performance of the male groups differed significantly on session 2 - trial 2 (†*p*  =  0.006), although large differences were also observed on session 1 - trial 2 (#*p*  =  0.049), and session 3 - trial 2 (#*p*  =  0.041).

At one month of age, results from the 1h locomotor activity test showed that the *Gnptab^−/−^* mice performed similarly to WT control mice in terms of general ambulatory activity, vertical rearing frequency, distance traveled in the periphery, and measures of emotionality including distance traveled, time spent, and entries made into a central zone in the test field (data not shown). In contrast, the *Gnptab^−/−^* mice exhibited impairment on 3/7 measures within the battery of sensorimotor tests. Specifically, significant genotype effects derived from ANOVAs conducted on these data revealed the following performance deficits in the *Gnptab^−/−^* mice: 1) *Gnptab^−/−^* mice remained on the elevated platform for a significantly shorter period of time (Fig. 1A) compared to the WT control mice (*p*  =  0.026); 2) the *Gnptab^−/−^* mice took significantly longer to climb down the pole (Fig. 1B) relative to WT mice (*p*  =  0.0007); and 3) the *Gnptab^−/−^* mice were able to remain upside down on the inverted screen for significantly less time (Fig. 1C) versus the WT controls (*p*  =  0.020; See Table S1 for all *F* statistics associated with these significant effects). No significant effects involving genotype were found for the ledge (Fig. 1D), walking initiation, or 60° and 90° inclined screen tests (data not shown). When tested on the sensorimotor battery at 4–5 months of age, a similar pattern of results was found, although the performance deficits of the *Gnptab^−/−^* mice were generally worse at this later age, typically resulting in larger differences between the groups (Fig. 1E–H). Besides a significant genotype effect, a significant sex effect (*p*  =  0.025) and genotype x sex interaction (*p*  =  0.041) were also revealed by the ANOVA conducted on the ledge data (Table S1). Subsequent contrasts showed that the male *Gnptab^−/−^* mice were significantly impaired on the ledge test compared to the male WT mice (*p*  =  0.002), while differences were not significant between the females of each group (Fig. 2A). No significant main or interaction effects involving sex were found for any of the other sensorimotor measures.

The *Gnptab^−/−^* mice also showed mildly impaired performance on some components of the rotarod test at 1 month of age (Fig. 1I–K). A repeated measures ANOVA (rmANOVA) revealed significant genotype (*p*  =  0.002) and sex (*p*  =  0.026) effects along with a significant genotype by trials interaction (*p*  =  0.0004) for the stationary rod condition (Fig. 1I; Table S1). Subsequent pair-wise comparisons showed that the *Gnptab^−/−^* mice spent significantly less time on the stationary rod compared to WT control mice during trial 1 (*p*  =  0.0007; Table S1). The *Gnptab^−/−^* mice were also significantly impaired relative to the WT control mice on the constant speed rotorod, but a significantly genotype x sessions interaction (p = 0.024) showed that this depended on the test session, with the greatest differences found during session 1 (Fig. 1J). No significant effects involving genotype were found for the accelerating rotarod data at 1 month of age. The performance of the *Gnptab^−/−^* mice declined with age such that robust (and significant) differences were found relative to the WT group for every trial across test sessions for each component of the rotarod procedure (Fig. 1L–N; Table S1) when the mice were retested at 4–5 months of age.

### *Gnptg^−/−^* mice have mild-to-moderate sensorimotor deficits

**Figure 3:**
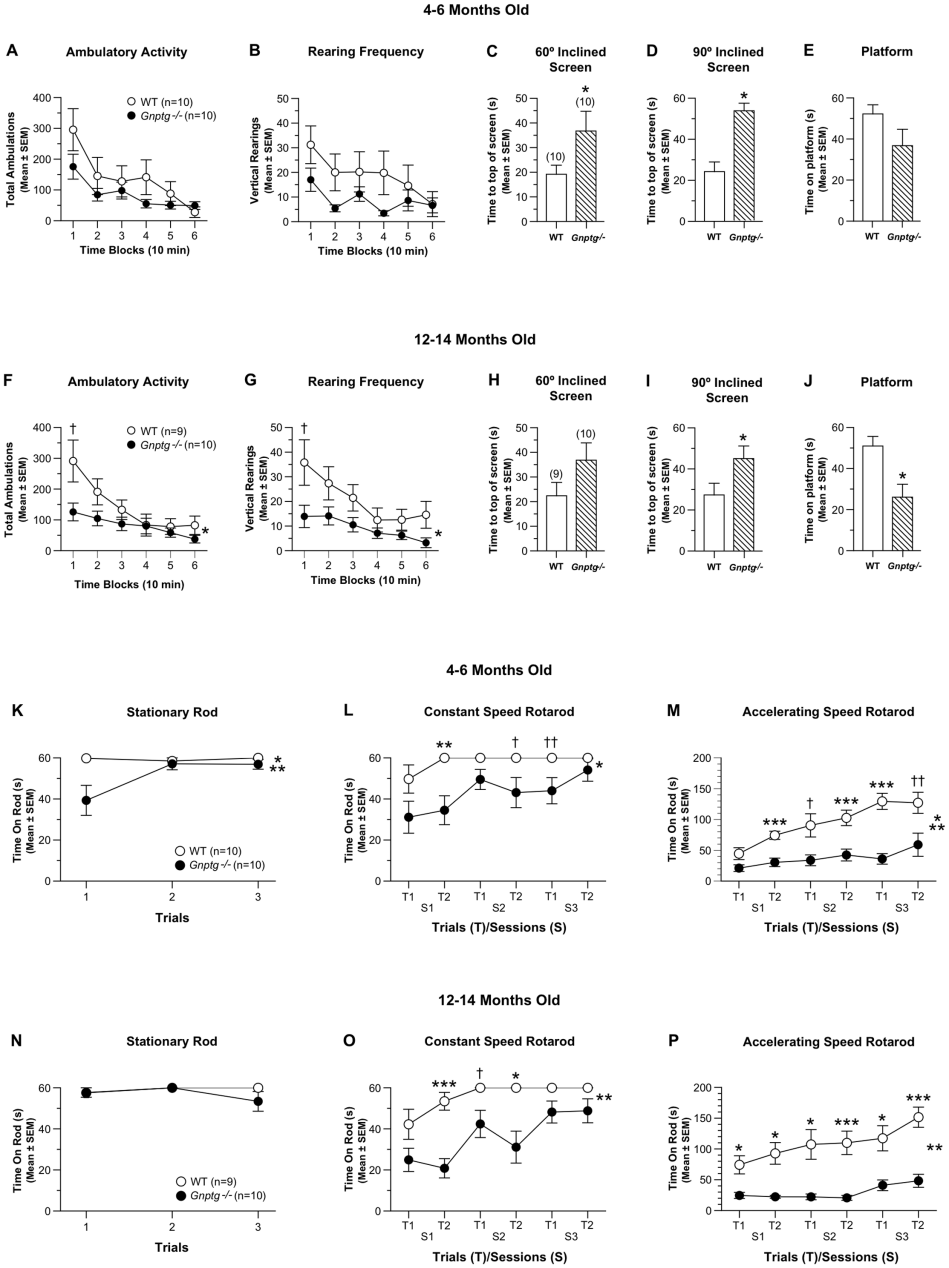
*Gnptg^−/−^* mice show mild to moderate performance deficits on activity, sensorimotor and rotarod tests. (A-B) Although the *Gnptg^−/−^* mice tended to show lower levels of ambulatory activity (A) and vertical rearing frequency (B) relative to WT littermate controls on the 1-h locomotor activity test when tested at 4–6 months of age, no significant effects involving genotype were found following analyses conducted on these data. However the *Gnptg^−/−^* group took significantly longer to climb to the top of the 60° (C) and 90° (D) inclined screens, (genotype effects: *p  =  0.0005 and 0.0001, respectively), and showed a nonsignificant trend toward being able to remain on the platform for a shorter time compared to WT control mice (E) when tested at this age (see Fig. 3B for details of sex effects on the 60° inclined screen). (F) When the 1-h activity test was conducted at 12–14 months of age, the *Gnptg^−/−^* mice tended to be less active compared to controls, although this depended on the time block of the test session (genotype x time interaction: *p  =  0.012), with the largest group differences being observed during the first time block (†*p*  =  0.041). (G) At this age, the *Gnptg^−/−^* group also showed significantly reduced rearing frequency on average across time blocks, (genotype effect: *p  =  0.043), with the largest differences occurring during the first block (†*p*  =  0.049). (H) During testing at 12-14 months of age, the performance level of the *Gnptg^−/−^* mice tended to be much lower than that of the WT control group on the 60° inclined screen, but the differences were not statistically significant. However, the *Gnptg^−/−^* mice did show significant performance deficits at this age on the 90° inclined screen (I) and platform tests (J), (genotype effects: *p  =  0.026 and *p  =  0.010, respectively). The *Gnptg^−/−^* strain was also impaired on the rotarod when tested at 4–6 months of age. (K) For example, a significant genotype effect (*p  =  0.024) indicated that the *Gnptg^−/−^* mice were impaired on the stationary rod component of the rotarod although this effect was mostly due to differences observed during trial 1 (††*p*  =  0.007), whereas their performance on the other two trials were similar to those of the WT group (see Fig. 3C for details of sex effects). (L) The *Gnptg^−/−^* group also exhibited significant performance impairments on the constant speed rotarod on average across trials and sessions (genotype effect: *p  =  0.005), with group differences being significant for session 1 - trial 2 (**p  =  0.004), while large differences were also observed for session 2 - trial 2 (†*p*  =  0.044) and session 3 - trial1 (††*p*  =  0.019). (M) Analysis of the accelerating rotarod data also revealed significant performance deficits on the part of the *Gnptg^−/−^* mice (genotype effect: **p  =  0.0003; genotype x sessions interaction: *p  =  0.002), although this was somewhat dependent on the sessions variable. Pair-wise comparisons showed that group performances differed significantly for session 1 - trial 2, session 2 - trial 2, and session 3 - trial 1 (***p <0.0009), while large differences were observed for session 2 - trial 1 (†*p*  =  0.012) and session 3 - trial 2 (††*p*  =  0.009). (See Fig. 3D for details concerning sex effects.) When the mice were re-tested at 12-14 months, the groups were found to perform similarly on the stationary rod (N). However, the *Gnptg^−/−^* group again showed significant deficits on the constant speed rotarod (O) which was documented by a significant genotype effect (**p  =  0.0003). Pair-wise comparisons showed that the *Gnptg^−/−^* mice had significantly reduced times on the rod for session 1 - trial 2 (***p  =  0.0003) and session 2 - trial 2 (*p  =  0.004), with large differences also being found for session 2 - trial1 (†*p*  =  0.033). (P) The *Gnptg^−/−^* group was also significantly impaired again on the accelerating rotarod when re-tested at the later age, (genotype effect: **p  =  0.0001) when pair-wise comparisons showed significant group differences across all trials and sessions (*p <0.008; ***p <0.0005) (see Fig. 3E for details of sex effects).

Analyses of the data from the 1h locomotor activity test of *Gnptg^−/−^* mice and the corresponding WT control group carried out at 4–6 months of age showed that the two groups performed similarly in terms of general ambulatory activity (Fig. 3A), vertical rearing (Fig. 3B), distance traveled in the periphery, and with regard to the emotionality (center) variables (not shown). The two groups also did not differ in several components of the sensorimotor battery, including walking initiation and the ledge, pole, and inverted screen tests (not shown). However, the *Gnptg^−/−^* mice exhibited significant performance deficits on the 60° and 90° inclined screen tests (Fig. 3C–D) compared to the WT mice (genotype effects: *p*  =  0.0005 and 0.0001, respectively; Table S2). The ANOVA conducted on the 60° inclined screen data also yielded a significant genotype x sex interaction, (*p*  =  0.041), with impaired performance being found for the male *Gnptg^−/−^* mice compared to the males from the WT group (*p*  =  0.0003), while the performance of the females from the two groups did not differ significantly (Fig. 2B). The *Gnptg^−/−^* group also tended to perform poorly on the platform test relative to the WT control mice, but these differences were not significant at this age (Fig. 3E). The data from the 1h locomotor activity test conducted when the mice were 12–14 months of age showed that the ambulatory activity and vertical rearing frequency of the *Gnptg^−/−^* mice were generally reduced relative to the WT littermates during the initial stages of the test (Fig. 3F–G;

Table S2). In addition, the *Gnptg^−/−^* group exhibited decreased vertical rearing frequency compared to the WT control mice (Fig. 3G; *p*  =  0.043; Table S2), with differences between the groups being greatest during block 1 (*p*  =  0.049). Similar to the activity results from the earlier age, no differences were observed between groups with regard to distance traveled in the periphery, or the emotionality variables (not shown). Although the *Gnptg^−/−^* mice showed a strong trend towards inferior performance at 12–14 months compared to WT littermate control mice with regard to the 60° inclined screen test, the differences were not significant like they were at the earlier time point (Fig. 3H). However the *Gnptg^−/−^* mice again showed a significant performance deficit relative to the WT group on the 90° inclined screen and also that the older mutant mice were impaired on the platform test (Fig. 3I–J; *p*  =  0.026 and 0.010, respectively; Table S2). Again, no group differences were observed on the walking initiation, ledge, platform or pole tests at the 12–14 month testing (not shown).

The *Gnptg^−/−^* mice also exhibited impaired performance on the rotarod test that was conducted when the mice were 4–6 months of age. They performed poorly during the first trial of the stationary rod component compared to the littermate WT group, but then the two groups performed comparably for the next two trials (Fig. 3K; Table S3). Additional contrasts showed that it was the female *Gnptg^−/−^* mice, not the males, who accounted for the impaired performance on trial 1 (Fig. 2C, Table S3). *Gnptg^−/−^* mice exhibited greater performance impairments on the constant speed rotarod (Fig. 3L) where they spent significantly less time on the rotarod on average across the trials and sessions (*p*  =  0.005; Table S3). The impaired performance of the *Gnptg^−/−^* mice was greatest during the accelerating rotarod test (Fig. 3M) where an rmANOVA yielded a significant genotype effect (*p*  =  0.0003) and significant genotype x sessions (*p*  =  0.002) and genotype x sex x trials (Fig. 2D; *p*  =  0.004) interaction (Table S3). When the mice were retested on the rotarod at 12–14 months of age, no performance differences were observed on the stationary rod (Fig. 3N) although large deficits were again observed on the part of the *Gnptg^−/−^* mice for the constant speed and accelerating rotarod components (Fig. 3O–P;Table S3).). The rmANOVA on these data also yielded a significant genotype x sex x trials x sessions interaction (*p*  =  0.017). Additional contrasts revealed that the female *Gnptg^−/−^* mice were significantly impaired on average across trials and sessions relative to the WT females (Fig. 2E; *p*  =  0.0001). Performance deficits were also observed in the male *Gnptg^−/−^* mice versus the male WT controls on average across trials and sessions, although the effect was smaller compared to that of the female groups (Fig. 2E; *p*  =  0.022).

### Histopathology

**Figure 4:**
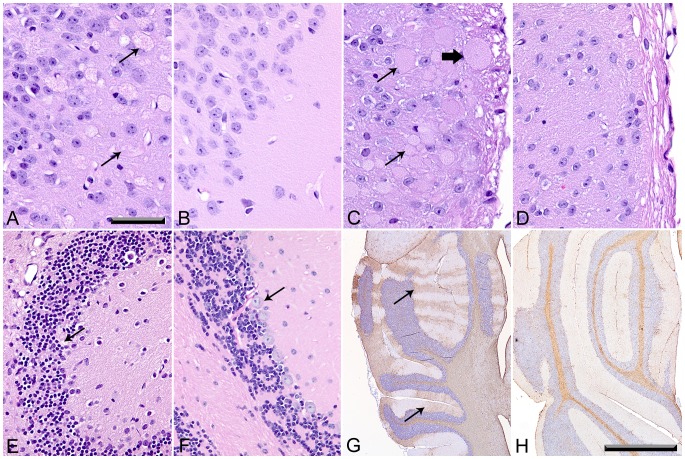
Histological lesions in H&E stained sections of brain and spinal cord were detected only in *Gnptab^−/−^* mice. (A) Swollen dystrophic axons/neurites in piriform cortex of 12-month-old *Gnptab^−/−^* mice, but absent in WT controls (B). (C) Axonal spheroids were present in the afferent nerves (thick arrow) and dystrophic neurites (thin arrows) were concentrated in the superficial laminae of the dorsal horn grey matter of the spinal cord of *Gnptab^−/−^* mice, but were entirely absent in the spinal cords of WT controls (D). (E) Cerebellum of 10/12-month-old *Gnptab^−/−^* mice with Purkinje cell loss (arrow) not seen in the Purkinje cell layer (arrow) in WT controls (F). (G) Staining for GFAP in cerebellum of 6-month old *Gnptab^−/−^* mice showing bilaterally symmetrical parasagittal bands (arrows) of activated Bergmann glial cells that coincided precisely with zones of Purkinje cell loss. (H) Negative GFAP staining of Bergmann's glia was evident in WT mice. (Figures A-F 40X, Bar  =  100 mm; Figures G and H; 4X, Bar  =  1 mm)

We initially used routine hematoxylin and eosin (H&E) stained sections of brain and spinal cord from WT, *Gnptab^−/−^* and *Gnptg^−/−^* mice to evaluate these tissues and then added periodic acid-Schiff (PAS) and immunohistochemistry (IHC) staining methods to more fully characterize the CNS lesions. Tissues were obtained from WT and *Gnptab^−/−^* mice at 4, 6, 10 and 12 months of age and from 12-month-old *Gnptg^−/−^* mice. At 4 months, no notable changes were seen in the CNS of WT or *Gnptab^−/−^* mice by routine H&E staining, but by 6 months of age, *Gnptab^−/−^* mice showed widely dispersed swollen dystrophic axons and neurites in multiple foci within the brain, the afferent spinal nerves and the dorsal horn grey matter of the spinal cord. The severity of clinical signs and the extent of associated histopathological lesions in *Gnptab^−/−^* mice continued to increase over time. In the 10- and 12-month-old *Gnptab^−/−^* mice dystrophic axons/neurites and spheroids were widely dispersed in the brainstem and dorsal grey matter of the spinal cord. These changes were particularly severe in the medial piriform (olfactory) cortex (Fig. 4A–B), especially in the area tempestas and along the midline from the lateral septal nucleus through the medial septal nucleus to the optic chiasm. In the spinal cord, dystrophic axons were mainly located within the dorsal nerve root, dorsolateral fasciculus, and dorsal root entry zone, whereas dystrophic neurites were concentrated in the superficial laminae of the dorsal horn grey matter (Fig. 4C–D). In both ascending and descending white tracts of the spinal cord there was diffuse vacuolization and rarefaction, with occasional axonal spheroids in 10- and 12-month-old *Gnptab^−/−^* mice. In addition, 10- and 12-month-old *Gnptab^−/−^* mice showed multifocal depletion of Purkinje cells in the cerebellum (Fig. 4E–F). Staining for GFAP in cerebellum of 6-month old *Gnptab^−/−^* mice showed bilaterally symmetrical parasagittal bands of activated Bergmann glial cells that coincided precisely with zones of Purkinje cell loss (Fig. 4G–H).

In marked contrast to the widespread pathological findings observed in *Gnptab^−/−^* mice, *Gnptg^−/−^* mice which do not display any abnormal clasping behavior did not develop any of the swollen dystrophic axons/neurites or white tract lesions that were widespread in the brains and spinal cords of age-matched *Gnptab^−/−^* mice. In fact, no CNS lesions of any type were detected in *Gnptg^−/−^* mice when using only H&E stained sections of the brain and spinal cord. However, as shown later, special staining methods used to study the tissues in more detail did reveal mild multifocal lesions in *Gnptg^−/−^* mice, but these were much more extensive in *Gnptab^−/−^* mice.

**Figure 5:**
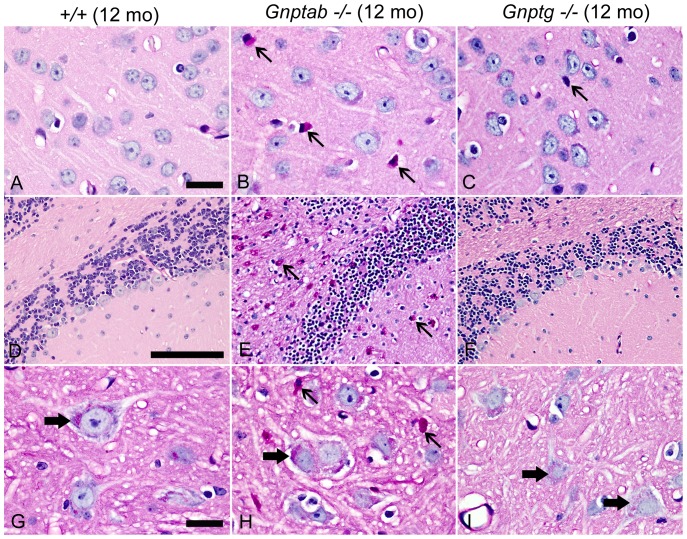
CNS tissue stained by the Periodic acid-Schiff method. (A) The CNS of 12-month-old WT mice lacked PAS-positive macrophages and microglia. (100X; Bar  =  20 mm) (B) 12-month-old *Gnptab^−/−^* mice exhibited widely dispersed enlarged PAS-positive microglia/macrophages (thin arrows) throughout most areas of brain and spinal cord. (C) PAS-positive microglia in *Gnptg^−/−^* mice were rare and small (thin arrow). (D) PAS-positive cells were absent in the white tracts and molecular layers of the WT cerebellum, abundant (thin arrows) in *Gnptab^−/−^* cerebellum (E), and absent in *Gnptg^−/−^* cerebellum (F). (G) Some of the larger neurons in brain and spinal cord of 12-month-old WT mice contained perinuclear clusters of very small, sharply defined PAS-positive granules. Age-matched *Gnptab^−/−^* mice (H), had markedly increased amounts of paler-staining granular material in the perinuclear cytoplasm of numerous large and medium size neurons (thick arrow) in addition to enlarged microglia/macrophages (thin arrows). The *Gnptg^−/−^* mice had low numbers of the pale-pink granular cytoplasmic material (thick arrows) (I).

CNS tissues of 12-month-old *Gnptab^−/−^* mice stained by the PAS method revealed widely dispersed PAS positive granular staining in macrophages and microglia throughout most areas of brain and spinal cord (Fig. 5B). Most of the strongly PAS-positive material was clearly cytoplasmic and restricted to microglia and macrophages. These changes were not present in the CNS of age-matched WT controls and were rare in *Gnptg^−/−^* mice (Fig. 5A/C). Genotype related differences in PAS staining were particularly evident within the white tracts and molecular layers of the cerebellum (compare Fig. 5E to 5D/F). These panels also show the selective loss of Purkinje cells in the *Gnptab^−/−^* tissue. Some of the larger neurons in both brain and spinal cord of the 12-month-old WT mice contained perinuclear clusters of very small and sharply defined PAS-positive granules, which likely represent lipofuscin (Fig. 5G). However, age-matched *Gnptab^−/−^* mice had markedly increased amounts of larger and paler-staining granular material in the perinuclear cytoplasm of numerous large and medium size neurons (Fig. 5H). Although similar pale-pink granular material was seen in some neurons of *Gnptg^−/−^* mice, it was present in smaller amounts and in far fewer neurons than in age-matched *Gnptab^−/−^* mice (Fig. 5I).

**Figure 6:**
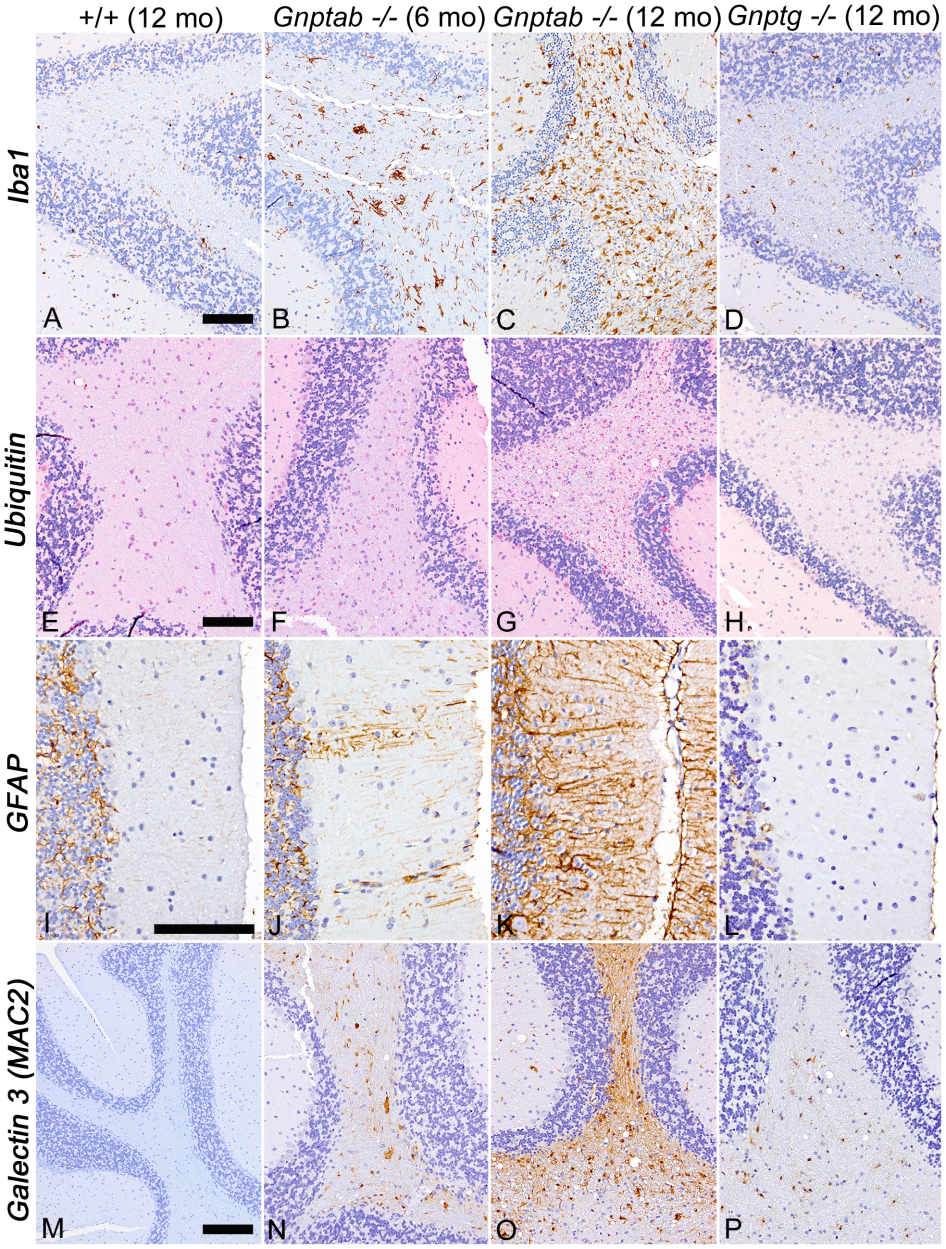
Cerebellar staining for *ionized-calcium binding protein 1* (Iba1), *ubiquitin* (UBQ), *glial fibrillary acidic protein* (GFAP), and *galectin-3* (MAC2) to detect microglia, ubiquinated proteins, astrocytes, and inflammation respectively. (A-D), IHC staining for Iba1 showed small microglia with delicate cytoplasmic extensions in 12 month old WT tissue (A), diffuse reactive microgliosis In 6-month-old *Gnptab^−/−^* mice (B), markedly increased staining in 12-month-old *Gnptab^−/−^* mice (C), and only mild activation of microglia in 12-month-old *Gnptg^−/−^* mice (D). (E) Ubiquitin-positive granules were rarely detected in cerebellar white tracts of 12-month-old WT mice. (F,G) The amount and extent of ubiquitin-positive granules increased in severity between 6 and 12 months of age in *Gnptab^−/−^* mice. (H) There was a minimal increase in ubiquitin staining in 12-month-old *Gnptg^−/−^* mice. (I-L) IHC staining for GFAP showed faint staining of Bergmann glial cells in 12-month-old WT mice (I), mild multifocal activation of Bergmann glial cells in 6 month old *Gnptab^−/−^* mice (J), bilaterally symmetrical areas of Purkinje cell loss with associated thinning of the molecular layer and diffuse activation of Bergmann glia in 12-month-old *Gnptab^−/−^* mice (K) and no notable change in GFAP staining patterns involving Bergmann glia in 12-month-old *Gnptg^−/−^* mice (L). (M-P) IHC staining of galectin-3 (MAC2) was negative in the CNS tissues of WT mice (M), but showed progressive staining of cerebellar peduncles and white tracts of 6 and 12 month old *Gnptab^−/−^* mice (N,O). MAC2 reactivity was mild and mostly restricted to the white tracts and peduncles of the cerebellum in 12 month old *Gnptg^−/−^* mice (P). Figures A - H and M - P, 20X; Bar  =  100 mm); Figures I - L, 40X; Bar  =  100 mm).

CNS tissues from the various types of mice were stained for *ionized-calcium binding protein* (Iba1), *ubiquitin* (UBQ), and *glial fibrillary acidic protein* (GFAP) in order to detect microglia, ubiquinated proteins, and astrocytes respectively. These staining methods served to highlight the true extent and progression of lesions in *Gnptab^−/−^* and *Gnptg^−/−^* mice. For example, staining for Iba1 showed no evidence of increased labeling in 12-month-old WT mice (Fig. 6A) but some Iba1-positive reactive microglia were detectable in the CNS of 4-month-old *Gnptab^−/−^* mice and by 6 months Iba1 staining of *Gnptab^−/−^* CNS showed diffuse moderate activation of microglia throughout the brain and spinal cord (Fig. 6B). The extent and severity of reactive microgliosis continued to increase in 12-month-old *Gnptab^−/−^* mice (Fig. 6C). In contrast to the widespread inflammatory and degenerative changes detected by IHC staining methods in 12-month-old *Gnptab^−/−^* mice, we found that CNS lesions in age-matched *Gnptg^−/−^* mice were extremely limited in extent and severity. Only mild reactive microgliosis was present in 12-month-old *Gnptg^−/−^* mice (Fig. 6D). Similarly, ubiquitin-positive granules were rarely detected in the white tracts of 12-month-old WT mice (Fig. 6E) but the amount and extent of ubiquitin-positive granules in *Gnptab^−/−^* mice increased in severity between ages 6 and 12 months (Fig. 6F, 6G). Although granular ubiquitin inclusions were abundant in the older *Gnptab^−/−^* mice in white tracts (especially with the corpus callosum and cerebellum) and dorsal sensory grey matter of the spinal cord, there was no significant labeling of either reactive microglia or neurons for ubiquitin. In contrast, there was only a minimal increase in ubiquitin staining in 12-month-old *Gnptg^−/−^* mice (Fig. 6H). The GFAP staining showed increased reactive astrogliosis present throughout the brain and spinal cord of the 6- and 12-month-old *Gnptab^−/−^* mice, with the progression of this lesion most clearly demonstrated in the molecular layer of the cerebellum. Whereas IHC staining for GFAP in this area showed only faint staining of Bergmann glial cells in WT mice (Fig. 6I), by 6 months of age *Gnptab^−/−^* mice showed bilaterally symmetrical parasagittal bands of activated Bergmann glial cells that coincided precisely with zones of Purkinje cell loss (Fig. 4G, 6J). The Purkinje cell loss and associated thinning of the molecular layer as well as the activation of associated Bergmann glia were much more prominent in 10- and 12-month-old *Gnptab^−/−^* mice (Fig. 6K). Except for some small areas of mild reactive astrogliosis in the cerebellar peduncles and white tracts, no notable changes in GFAP staining patterns were seen in the brain or spinal cord of 12-month-old *Gnptg^−/−^* mice (Fig. 6L). Another indicator of the progressively increased severity of CNS inflammation in *Gnptab^−/−^* mice was demonstrated by the appearance of galectin-3 (MAC2: a marker for inflammation) staining in the white tracts in both brain and spinal cord. WT mice were essentially negative for galectin-3 staining (Fig. 6M) but cerebellar white tracts, trigeminal nerves, and dorsal/dorsolateral white tracts of the spinal cord in 6-month-old *Gnptab^−/−^* mice were labeled for this marker (Fig. 6N). The extent and severity of labeling increased in proportion to the amount of nerve damage and by 12 months of age *Gnptab^−/−^* mice showed diffuse staining of cerebellar peduncles and white tracts in the cerebellum (Fig. 6O) and widespread staining of ventrolateral fasciculi in the spinal cord. The much milder MAC2 reactivity in 12-month-old *Gnptg^−/−^* mice was also primarily found in the white tracts and peduncles of the cerebellum (Fig. 6P) although small foci were also labeled in spinal cord white tracts.

**Figure 7:**
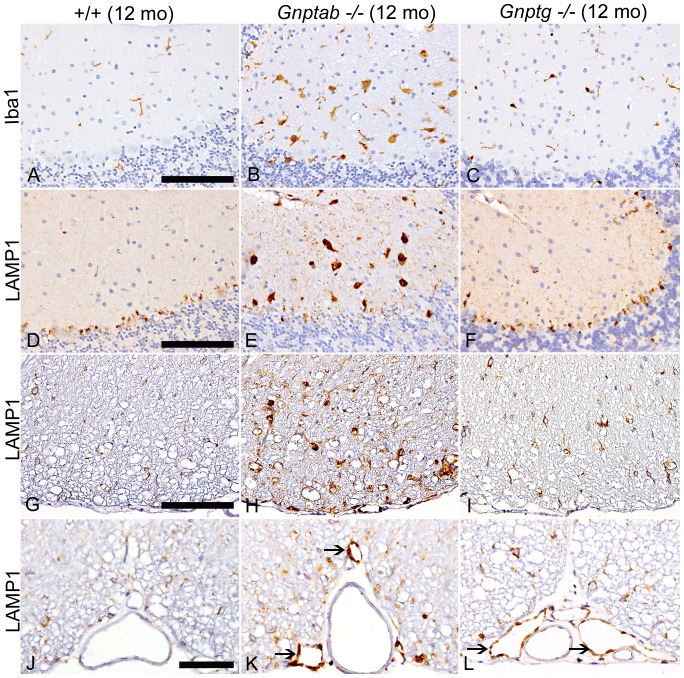
CNS staining for ionized-calcium binding protein 1 (Iba1) and lysosomal-associated membrane protein1 (LAMP-1). IHC staining of the cerebellum for Iba1 (A-C) and LAMP-1 (D-F). (A) Molecular layer of cerebellum in 12-month-old WT mice showing nonreactive resident microglia characterized by fine cytoplasmic extensions. (B) In 12-month-old *Gnptab^−/−^* mice, there are numerous enlarged Iba1/MAC2 positive macrophages in the molecular layers associated with Purkinje cell loss. (C) In 12-month-old *Gnptg^−/−^* mice, there was only mild activation of microglia in the molecular layer. (D) LAMP-1 in 12-month-old WT cerebellum is limited primarily to the Purkinje cell layer. (E) In contrast, LAMP-1 labeling in 12-month-old *Gnptab^−/−^* mice is prominent in the enlarged macrophages/microglia in the molecular layer but reduced in the areas of Purkinje cell loss. (F) There was a slightly increased amount of LAMP-1 staining in the Purkinje cell and molecular layers of 12-month-old *Gnptg^−/−^* mice, suggesting a subclinical increase in lysosomal storage in these areas. In (G-I) similar genotype-related differences in LAMP-1 staining are shown in spinal cord white tracts in 12-month-old WT, *Gnptab^−/−^* and *Gnptg^−/−^* mice, respectively. In all genotypes LAMP-1 was detected within oligodendrocytes, but the extent of labeling was markedly increased in *Gnptab^−/−^* mice (H) due to increased volume of cytoplasm and extensions of hypertrophic oligodendrocytes, as well as reactive microglia and macrophages. (I) In 12-month-old *Gnptg^−/−^* mice, LAMP-1-positive oligodendrocytes are also larger and more prominent than in WT mice, but microglia/macrophages are uncommon. (J-L) Endothelial cells in meningeal veins in WT mice were consistently LAMP-1 negative (J), but microvesiculated endothelium of meningeal veins (not arteries) in both *Gnptab^−/−^* (K) and *Gnptg^−/−^* (L) mice was LAMP-1 positive, although markedly more so in the *Gnptab^−/−^* mice. (Figs. A - I at 40X,Bar  =  100 mm; Figs. J - L at 60X, Bar  =  50 mm)

In addition to the increased labeling by Iba1 and MAC2 of cells found in almost all areas of the brain and spinal cord of *Gnptab^−/−^* mice, there was enhanced LAMP-1 *(Lysosomal-associated membrane protein 1)* staining in these areas, indicative of lysosomes distended by undigested material. The LAMP-1 labeling that is evident in many neurons of the *Gnptab^−/−^* mice (Fig. 7E) was also mildly increased in *Gnptg^−/−^* mice in comparison to WT controls (Fig. 7F vs. 7D). In the molecular layer of the cerebellum in WT mice, Iba1 labels non-reactive resident microglia (Fig. 7A) but no MAC2 staining is evident (Fig. 6A). However, in *Gnptab^−/−^* mice there are numerous enlarged Iba1/MAC2 positive macrophages (Fig. 6O, 7B) in the molecular areas showing loss of Purkinje cells. Only mild activation of microglia is evident in the molecular layer in 12-month-old *Gnptg^−/−^* mice (Fig. 7C).

Of note, the LAMP-1 staining in WT cerebellum is limited primarily to the Purkinje cell layer (Fig. 7D). In contrast, LAMP-1 labeling in *Gnptab^−/−^* mice is prominent in the enlarged macrophages/microglia in the molecular layer but is reduced in the areas of Purkinje cell loss (Fig. 7E). Although Purkinje cell loss and hypertrophy of phagocytic cells are not seen in the molecular layer of 12-month-old *Gnptg^−/−^* mice, there is an increased amount of LAMP-1 staining in the Purkinje cell and molecular layers (Fig. 7F), suggesting that there is a subclinical increase in lysosomal storage in these areas. The markedly increased extent of LAMP-1 staining of perinuclear cytoplasmic granules in neurons in *Gnptab^−/−^* mice indicates greater lysosomal storage in neurons in comparison to WT and *Gnptg^−/−^* mice.

Similar differences in LAMP-1 staining were noted in the spinal cord white tracts in WT, *Gnptg^−/−^* and *Gnptab^−/−^* mice. In all mice, LAMP-1 was detected within oligodendrocytes, identified based on morphology, location in white tracts and lack of staining for MAC2 and Iba1. However, the extent of labeling was markedly increased in *Gnptab^−/−^* mice due to the more extensive cytoplasmic volume and extensions of reactive microglia and hypertrophic oligodendrocytes (Figs. 7G,H,I). It was also noted that endothelial cells in WT mice were LAMP-1 negative but that the microvesiculated endothelium of meningeal veins (not arteries) in *Gnptab^−/−^* and *Gnptg^−/−^* mice was LAMP-1 positive although markedly more so in the *Gnptab^−/−^* mice (Figs. 7J,K,L).

**Figure 8:**
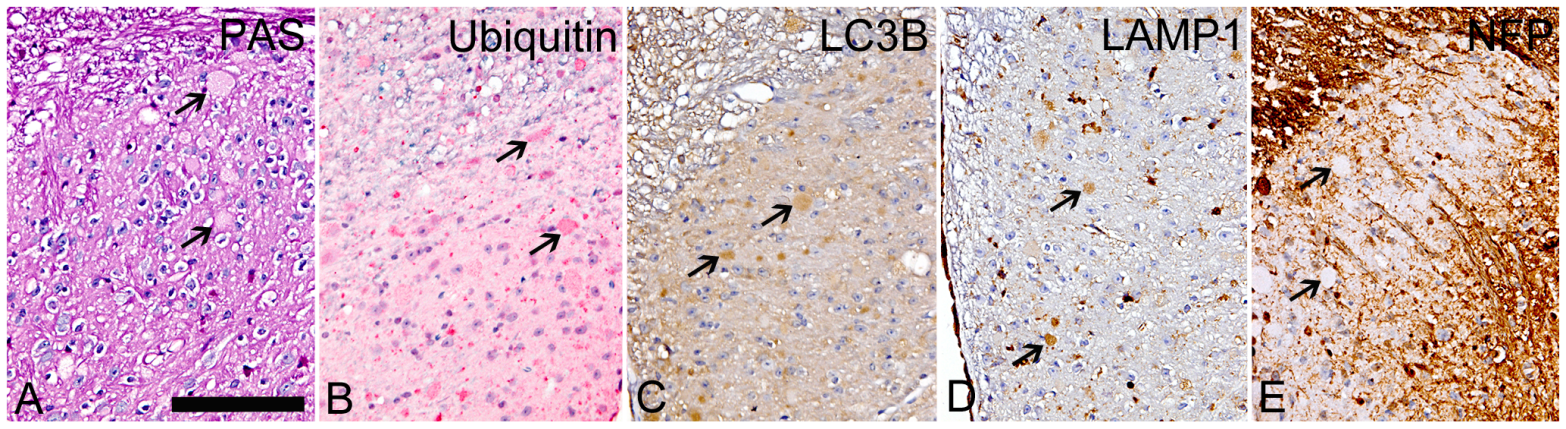
PAS and IHC staining of brain and spinal cord dystrophic axons and neurites of 12-month-old *Gnptab^−/−^* mice. (A) The dystrophic axons and neurites were consistently negative for PAS (arrows). (B) There was widespread granular staining of most dystrophic axons/neurites for ubiquitin (arrows). In contrast, IHC staining for the autophagy marker LC3B (C) and the lysosome/endosome marker LAMP-1 (D) showed variable (negative to mild) staining of dystrophic axons/neurites (arrows). (E) Strong staining for *neurofilament protein* (NFP) was present in only a few dystrophic axons in the white tracts of the cerebellum and spinal cord of *Gnptab^−/−^* mice, while the abundant large swollen neurites in the superficial laminae (layers 1 to 3) of the dorsal horn grey matter were consistently NFP-negative (arrows). (40X; Bar  =  100 mm)

Finally, we used PAS staining and selected IHC stains to better characterize the nature/origin of the swollen dystrophic axons/neurites, which were seen only in the brain and spinal cord of the *Gnptab^−/−^* mice. Notably, the numerous dystrophic axons and neurites in 10-12-month-old *Gnptab^−/−^* mice were consistently negative for PAS (Fig. 8A). The staining of the dystrophic axons/neurites for the other markers was highly variable. For example, there was widespread granular staining of most dystrophic axons/neurites for ubiquitin (Fig. 8B) while IHC staining for the autophagy marker LC3B (Fig. 8C) and the lysosome/late endosome marker LAMP-1 (Fig. 8D) showed variable (negative to mild) staining. In contrast, strong staining for neurofilamen*t protein* (NFP) was present in only a few dystrophic axons in the white tracts of the cerebellum and spinal cord of *Gnptab^−/−^* mice while the abundant large swollen neurites in the superficial laminae (layers 1 to 3) of the dorsal horn grey matter were consistently NFP-negative (Fig. 8E) with only a few bodies scattered in deeper laminae (layers 4-9) being NFP-positive. Taken together, these staining results were indeterminate, but suggest that the bodies in the swollen dystrophic axons/neurites may consist of a mixture of distended autophagosomes and autolysosomes.

### Analysis of CNS storage material

**Figure 9:**
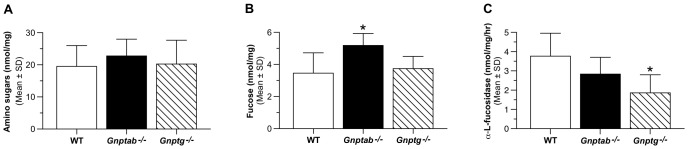
*Gnptab^−/−^* mice have an accumulation of fucosylated oligosaccharides in the brain. One-year-old WT, *Gnptab^−/−^* or *Gnptg^−/−^* mice were perfused with PBS and CNS material harvested and frozen until analyzed. Levels of amino sugars (A), or fucosylated glycans was determined (B) (data plotted as mean ±SD, n = 3-5, *P<0.05). (C) Six months – 1 year old WT, *Gnptab^−/−^* or *Gnptg^−/−^* mice were perfused with PBS and whole brain lysates were used to determine the activity of α-L-fucosidase (data plotted as mean ±SD, n = 6-10. All data was analyzed using an unpaired Student's t-test.

The findings of PAS positive material in the CNS of 10- and 12-month-old *Gnptab^−/−^* mice indicated the presence of carbohydrate-containing storage material. To better characterize the nature of the carbohydrate accumulation found in the CNS of *Gnptab^−/−^* mice, we determined the content of total amino sugars and fucose in the brains of the mutant mice. These assays showed a small accumulation of amino sugars in the CNS of 12-month-old *Gnptab^−/−^* mice that did not reach statistical significance (Fig. 9A) but a significant increase in the level of fucose (p<0.05) (Fig. 9B). In contrast, the level of these sugars was normal in the *Gnptg^−/−^* mice. Assay of whole brain extracts of WT or mutant mice for lysosomal α-L-fucosidase activity showed that the *Gnptab^−/−^* mice had a decreased level of this hydrolase that did not quite achieve significance (p = 0.0587) whereas the *Gnptg^−/−^* mice had a significant decrease in α-L-fucosidase activity (p = 0.0059) (Fig. 9C). One explanation for the lack of correlation between fucose accumulation and reduction in α-L-fucosidase activity is that these changes are not uniform among the various cell types in the brain and the assays of total brain tissue obscures changes in specific cell types.

**Figure 10:**
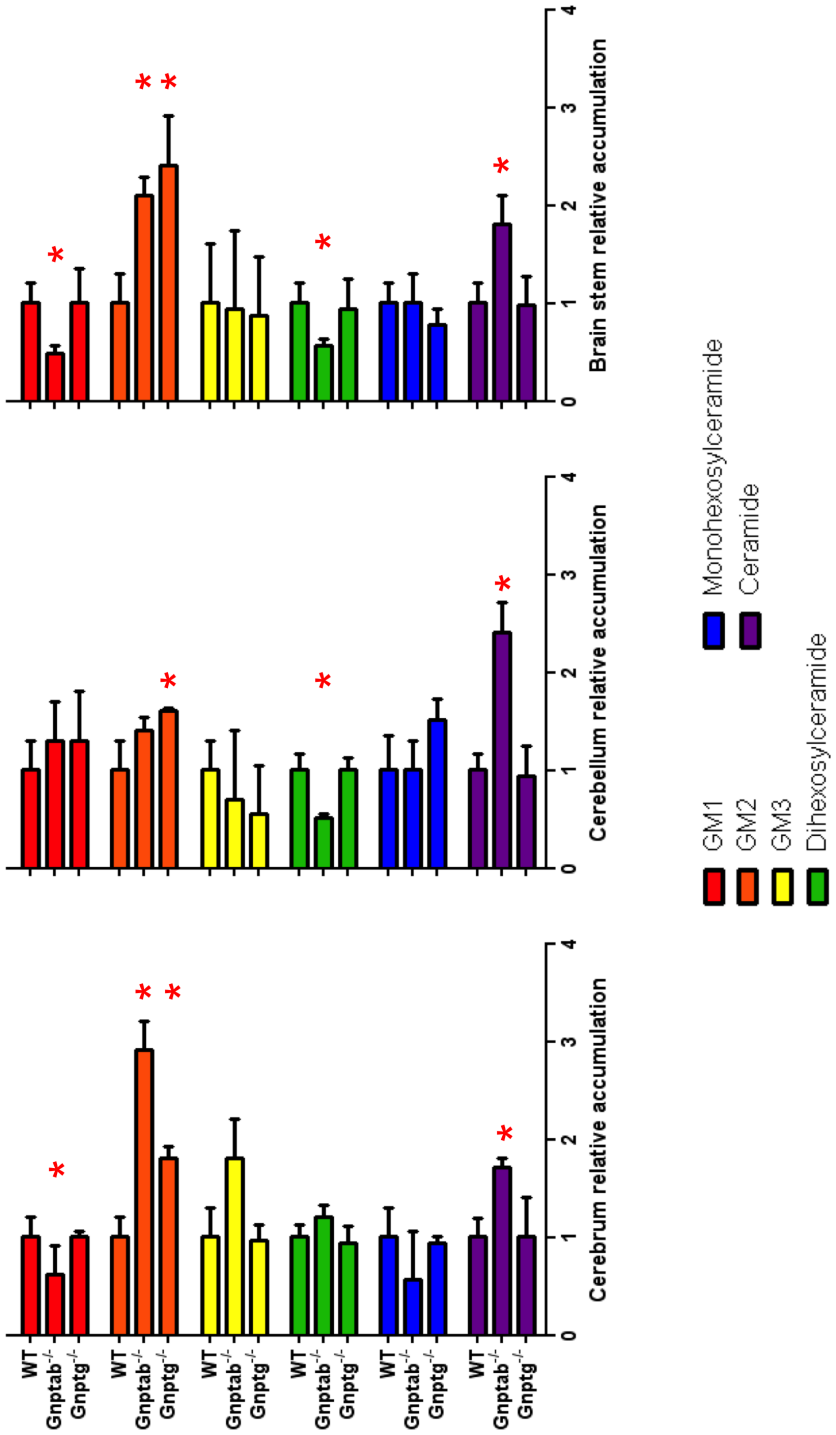
Analysis of Gangliosides and other lipids in *Gnptab^−/−^* and *Gnptg^−/−^* mice brains. Cerebrum, cerebellum or brain stem was isolated from 12-month-old WT, *Gnptab^−/−^* or *Gnptg^−/−^* mice and the isolated lipids analyzed by mass spectrometry. Each sample was analyzed in duplicate and normalized to protein content (ng species/mg total protein) with *n* = 6 mice for WT and n = 3 for *Gnptab^−/−^* and *Gnptg^−/−^* mice. Ratio of lipid species/mg protein for WT mice was set to 1 (mean ± SD, *p<0.05). Data shown represents the d18∶1, C18∶0 species for GM1, GM2 and GM3. The dihexosylceramide, monohexosylceramide, ceramide data represents the d18∶1, C16∶0 species. All data was analyzed using an unpaired Student's t-test.

Mass Spectrometry analysis of brain tissue from 12-month-old *Gnptab^−/−^* mice revealed an accumulation of GM2 in the cerebrum and brainstem while *Gnptg^−/−^* mice had accumulation of GM2 in the cerebrum, cerebellum and brainstem (Fig. 10). No significant changes were found in the level of GM3 and the level of GM1 showed a decrease in cerebrum and brainstem for the *Gnptab^−/−^* mice. The *Gnptab^−/−^* mice also had an accumulation of ceramide in all three regions of the brain and a reduction in dihexosylceramides in the brainstem and cerebellum. These data suggest that in some cells of the brain, the activites of α-L-fucosidase, β-hexosaminidase and likely other acid hydrolases are sufficiently deficient to allow the accumulation of undigested material in lysosomes.

## Discussion

In this study we used mouse models of ML II and ML III γ to compare and contrast the course of neurologic and behavioral changes associated with either complete or partial loss of the Man-6-P-targeting signal. We previously reported that both *Gnptab^−/−^* and *Gnptg^−/−^* mice had unremarkable neurologic exams up to 14 weeks with the exception of increased pain tolerance in the *Gnptab^−/−^* mice [9]. However, we subsequently observed that the *Gnptab^−/−^* mice developed a hind limb clasping response between 4-6 months. This prompted us to initiate more extensive behavioral and pathologic studies of the two mouse models. We found that the *Gnptab^−/−^* mice start exhibiting performance deficits on behavioral tests that assess sensorimotor functioning at 1 month of age, with progressive deterioration in performance being observed at 4 months. Results from the rotarod and pole tests suggest that the *Gnptab^−/−^* mice have impaired coordination between the forelimbs and the hindlimbs, although compromised strength and balance (inverted screen and platform test results) may contribute to this. Although differences in the experimental designs of the *Gnptab^−/−^* and *Gnptg^−/−^* studies make it inappropriate to directly compare the results between the two models, it appears that the *Gnptg^−/−^* mice generally showed less severe sensorimotor deficits suggesting more mildly disrupted sensorimotor functions than the *Gnptab^−/−^* strain and possibly less extensive neuropathology.

The more extreme behavioral disturbances in the *Gnptab^−/−^* mice were associated with progressive neurodegeneration characterized by microgliosis, astrocytosis, Purkinje cell loss, widely dispersed swollen dystrophic axons and neurites filled with granular material along with the accumulation of PAS- and ubiquitin-positive granules in various cell types. The changes were widespread, involving the spinal cord, brainstem, cerebrum and cerebellum. The dystrophic neurites and axons in the spinal cord of *Gnptab^−/−^* mice were concentrated in the dorsal horn and extended into the dorsal columns, suggesting that sensory neurons and pathways were affected. Lesions in the ventral and lateral regions of the spinal cord primarily involved the white tracts and were characterized by vacuolization of axons and hypertrophy of associated LC3B-positive oligodendrocytes. Some motor neurons contained increased amounts of PAS-positive material (lipofuscin?). Taken together, these findings suggest that subsets of both sensory and motor neurons were affected in *Gnptab^−/−^* mice, in contrast to the almost normal histopathology of the *Gnptg^−/−^* mice.

The hypertrophy and increased LAMP-1 staining of oligodendrocytes in areas of white tract vacuolization suggests that oligodendrocytes are particularly susceptible to the lysosomal enzyme deficiencies that occur in *Gnptab^−/−^* mice. Lesions involving both white tracts and neurons were widespread but not diffuse in both the brain and spinal cord, indicating that cell subsets in the CNS differ in their susceptibility to lysosomal enzyme deficiencies. Similar lesions were not present in gamma KO mice.

The immunohistochemical staining for GFAP in the *Gnptab^−/−^* cerebellum showed a striking pattern with the cellular processes of reactive Bergmann glial cells appearing as prominent parasagittal bands. Notably, these bands of reactive GFAP-positive Bergmann glia correlated directly with areas of Purkinje cell degeneration and loss, a pattern also seen in the brains of humans and mice with other lysosomal storage disorders, such as Juvenile neuronal ceroid lipofuscinosis [20]. The cerebellum is organized into genetically defined subdivisions that appear to form distinct functional units within the cerebellum [21]-[23]. Many other markers have shown similar staining patterns of parasagittal bands in normal cerebellum and the bands appear to correlate with different Purkinje cell subtypes having well-established patterns of gene expression [22], [24].

It is difficult to compare these findings to those reported for ML II patients since only a few autopsies have been reported and most of them did not include IHC stains. However, for both ML II patients and mice, macroscopic evaluation of the brain and spinal cord revealed little to no abnormalities, but the microscopic examination reported here showed evidence of storage material, vacuolization, Purkinje cell loss and positive PAS staining in neurons.

In contrast to the *Gnptab^−/−^* mice, the *Gnptg^−/−^* mice showed impaired performance on only a few of the behavioral tests and these abnormalities did not become evident until these mice were much older compared to *Gnptab^−/−^* mice (4-6 months versus 1 month), although more precise information regarding the age of onset of these behavioral deficits requires testing at earlier ages in the *Gnptg^−/−^* mice. Further, changes in the *Gnptg^−/−^* mouse brain were slower to develop and much milder in extent and severity than those seen in the *Gnptab^−/−^* mice. This correlates well with the finding that ML III γ patients have normal to mildly impaired neurologic functioning [25]-[27]. In the present study, we have also described a few genotype-dependent sex effects in the two strains of mutant mice. Presently, the significance of these findings for the two forms of mucolipidoses being modeled here is not clear, yet we have mentioned them to promote further research into possible sex effects in the human disorders.

It is interesting that in spite of finding that the activity of a number of acid hydrolases in total brain extracts of the *Gnptab^−/−^* and *Gnptg^−/−^* mice is normal or only moderately decreased and even increased in a few instances [10], [11], several types of storage materials were detected in the brains of the mutant mice, especially in the *Gnptab^−/−^* mice, consistent with defective lysosomal function. PAS positive material and increased fucose-containing glycans were found in the brains of older *Gnptab^−/−^* mice but not *Gnptg^−/−^* mice whereas both types of mutant mice showed accumulation of GM2 in their cerebrum and brainstem. However, it must be noted that secondary accumulation of GM2 has been reported in a number of lysosomal storage diseases [28]. *Gnptab^−/−^* mice also had an accumulation of ceramide in all three regions of the brain. Since accumulation of gangliosides has been associated with inflammation and neuronal degeneration [28], this abnormality could potentially be a contributing factor in the neurologic abnormalities present in the *Gnptab^−/−^* and *Gnptg^−/−^* mice. However, it seems unlikely that this ganglioside accumulation is the predominant factor in the development of the CNS pathology and behavioral alterations seen in the *Gnptab^−/−^* mice as GM2 accumulates to similar levels in the *Gnptab^−/−^* mice and the more mildly affected *Gnptg^−/−^* mice. Since we did not analyze individual subpopulations of cells in the brain, we cannot exclude the possibility that the GM2 accumulation is happening in different cells types or regions of the brain in the two models thus influencing the degree or type of impairment. However, a more likely explanation is the accumulation of different materials or different amounts of undigested material in the CNS of *Gnptab^−/−^* mice versus the *Gnptg^−/−^* mice.

While this work was in progress, Kollmann et al. [12] reported neurologic findings in a mouse model of ML II generated by knocking in a version of *Gnptab* containing a mutation originally identified in ML II patients, *Gnptab^c.3028insC^* [29]. In many respects the findings with the two models are similar although there are some differences. One concerns life span. The vast majority of our *Gnptab^−/−^* mice survive to 40 weeks and then either die or have to be euthanized due to disease progression by 60 weeks (data not shown). In contrast, the *Gnptab^c.3082insC^* mice have a steady decline in survival starting at birth with ∼50% of the mice dead prior to 40 weeks and only a few surviving up to 64 weeks [12]. Importantly, both models exhibit many similar neurologic abnormalities including prominent astrogliosis, activated microglia, swollen axons containing storage material in the cerebellum along with the loss of Purkinje cells. However, we do not find the reduction in cerebellar size reported in the *Gnptab^c.3082insC^* mice. This abnormality has also not been reported in ML II patient autopsies. The *Gnptab^c.3082insC^* mice also showed an accumulation of cholesterol in the Purkinje and granule layer of the cerebellum based on filipin staining [12] whereas we did not detect cholesterol accumulation by MS in either the *Gnptab^−/−^* or *Gnptg^−/−^* mice. This difference may be due to the methods used to detect cholesterol since filipin staining can detect alterations in the subcellular distribution of cholesterol that don't necessarily involve an increase in the total content of this compound. Finally, we have previously reported that the levels of LC3-II, LAMP-2 and ubiquitinated proteins in the brains of *Gnptab^−/−^* mice were unchanged as determined by western blotting [11]. This differs from the finding in the brain of *Gnptab^c.3082insC^* mice, which showed increased LC3-II and p62 using this technique. However, using IHC we did detect increased staining of ubiquitin-positive granules in white tracts, especially in the corpus callosum and cerebellum, and the dorsal sensory gray matter of the spinal cord at 6 months of age with considerable progression at 12 months of age.

The modest differences in the two models may be a result of several factors. One possibility is that since the *Gnptab^−/−^* mice were generated through an insertional mutagenesis strategy [8], some degree of splicing may occur around the gene-trap cassette, resulting in the generation of a small amount of GlcNAc-1-phosphotransferase that might be sufficient to target low levels of acid hydrolases to the lysosome. We previously reported that the acid hydrolases of the brain of *Gnptab^−/−^* mice had no detectable Man-6-P phosphorylation (less than 2% in these assays) whereas the same acid hydrolases of WT mice had 41-82% phosphorylation [2], [10]. Other possibilities behind the difference include mixed strain differences (*Gnptab^−/−^* mice in C57BL/6-129SvEvBrd versus *Gnptab^c.3082insC^* mice in C57BL/6-129SvJ) or secondary effects of residual C-terminally truncated α/β precursor protein in the *Gnptab^c.3082insC^* mice such as activation of the endoplasmic-reticulum-associated protein degradation (ERAD) system. Regardless of these differences in the two mouse models of ML II, the finding that both exhibit severe neurodegeneration confirms that the Man-6-P independent sorting pathway is unable to fully support adequate lysosomal function in the CNS. The more benign course of the *Gnptg^−/−^* mice shows that the partial phosphorylation of acid hydrolases is effective in decreasing the lysosomal dysfunction and lessening the associated neurodegeneration and behavioral changes.

### Addendum

While this manuscript was in revision, Paton *et. al.*, (Paton L, Bitoun E, Kenyon J, Periestma DA, Oliver PL et. al., (2014) A novel mouse model of a patient mucolipidosis II mutation recapitulates disease pathology. The Journal of biological chemistry, in press. Published on August 8, 2014) reported a new mouse model of ML II, characterized by growth retardation, skeletal and facial abnormalities and reduced life span. These mice exhibited impaired motor function and psychomotor retardation. Histological analysis showed progressive degeneration in the cerebellum. While many of the features are similar to those found in our mutant mice, our mice lack the craniofacial defects found in the new model and the very early onset of death.

## Supporting Information

Table S1 Note that one *Gnptab^−/−^* mouse that had an injured paw and one WT mouse that continually climbed onto the apparatus rather than remaining on the rod were dropped from the analyses of the rotarod data.
(PDF)

Table S2 Note that one WT mouse died between the 4–6 months old and 12–14 months old tests. Abbreviation: Incl.  =  Inclined
(PDF)

Table S3 Significant ANOVA effects involving genotype (Geno) and sex variables from the rotarod test in 4–6 and 12–14 months old Gnptg−/− and WT mice.
(PDF)
