# Correction: Neurologic Abnormalities in Mouse Models of the Lysosomal Storage Disorders Mucolipidosis II and Mucolipidosis III γ

**DOI:** 10.1371/journal.pone.0114199

**Published:** 2014-11-18

**Authors:** The PLOS ONE Staff

## Notice of Republication

This article was republished on October 28, 2014, due to multiple instances of "Gnptab-/-" being published as "Gnptg-/- ". The publisher apologizes for this error. Please download the article again to view the corrected version. The originally published, uncorrected article and the republished, corrected article are provided here for reference.

## Supporting Information

File S1 Originally published, uncorrected article
(PDF)

File S2 Republished, corrected article
(PDF)
