# Supplementary material for: Neurologic Abnormalities in Mouse Models of the Lysosomal Storage Disorders Mucolipidosis II and Mucolipidosis III γ
Source: PLoS One. 2014 Oct 14;9(10):e109768. doi: 10.1371/journal.pone.0109768 (PMC4196941; doi:10.1371/journal.pone.0109768)
Supplement: Table S1 — Note that one Gnptab−/− mouse that had an injured paw and one WT mouse that continually climbed onto the apparatus rather than remaining on the rod were dropped from the analyses of the rotarod data. (PDF) [file pone.0109768.s001.pdf]

Table S1. Significant ANOVA effects involving genotype (Geno) and sex variables from the sensorimotor battery and rotarod test in 1-month and 4-5 months old *Gnptab*$^{-/-}$ and WT mice.

Test (Age)	Effect	F Statistics
Sensorimotor Battery		
Platform (1 mo)	Geno	$F_{(1,24)}$=5.66, p=0.026
Pole (1 mo)	Geno	$F_{(1,24)}$=,15.15 p=0.0007
Inverted Screen (1 mo)	Geno	$F_{(1,24)}$=6.18, p=0.020
Platform (4-5 mos)	Geno	$F_{(1,23)}$=19.10, p=0.0002
Pole (4-5 mos)	Geno	$F_{(1,23)}$=16.59, p=0.0005
Inverted Screen (4-5 mos)	Geno	$F_{(1,23)}$=230.88, p<0.00005
Ledge (4-5 mos)	Geno	$F_{(1,23)}$=14.28, p=0.001
	Sex	$F_{(1,23)}$=5.74, p=0.025
	Geno x Sex	$F_{(1,23)}$=4.70, p=0.041
Rotarod		
Stationary (1 mo)	Geno	$F_{(1,22)}$=12.29, p=0.002
	Sex	$F_{(1,22)}$=5.70, p=0.026
	Geno x Trials	$F_{(2,44)}$=9.32, p=0.0004
	Trial 1	$F_{(1,22)}$=15.67, p=0.0007
Constant Speed (1 mo)	Geno x Sessions	$F_{(2,44)}$=4.04, p=0.024
	Session 1, Trial 1	$F_{(1,22)}$=4.48, p=0.046
	Session 1, Trial 2	$F_{(1,22)}$=6.03, p=0.022
Stationary (4-5 mos)	Geno	$F_{(1,21)}$=35.22, p<0.00005
	Trial 1	$F_{(1,21)}$=18.39, p=0.0003
	Trial 2	$F_{(1,21)}$=19.61, p=0.0002
	Trial 3	$F_{(1,21)}$=17.87, p=0.0004
Constant Speed (4-5 mos)	Geno	$F_{(1,21)}$=31.91, p<0.00005
	Session 1, Trial 1	$F_{(1,21)}$=12.83, p=0.002
	Session 1, Trial 2	$F_{(1,21)}$=14.16, p=0.001
	Session 2, Trial 1	$F_{(1,21)}$=38.80, p<0.00005
	Session 2, Trial 2	$F_{(1,21)}$=26.62, p<0.00005
	Session 3, Trial 1	$F_{(1,21)}$=16.21, p=0.0006
	Session 3, Trial 2	$F_{(1,21)}$=18.60, p=0.0003
Accelerating (4-5 mos)	Geno	$F_{(1,21)}$=38.65, p<0.00005
	Geno x Trials	$F_{(1,21)}$=8.15, p=0.010
	Session 1, Trial1	$F_{(1,21)}$=20.58, p=0.0002
	Session 1, Trial 2	$F_{(1,21)}$=33.70, p<0.00005
	Session 2, Trial 1	$F_{(1,21)}$=33.25, p<0.00005
	Session 2, Trial 2	$F_{(1,21)}$=28.75, p<0.00005
	Session 3, Trial 1	$F_{(1,21)}$=12.54, p=0.002
	Session 3, Trial 2	$F_{(1,21)}$=29.43, p<0.00005
