# Supplementary material for: Neurologic Abnormalities in Mouse Models of the Lysosomal Storage Disorders Mucolipidosis II and Mucolipidosis III γ
Source: PLoS One. 2014 Oct 14;9(10):e109768. doi: 10.1371/journal.pone.0109768 (PMC4196941; doi:10.1371/journal.pone.0109768)
Supplement: Table S2 — Note that one WT mouse died between the 4–6 months old and 12–14 months old tests. Abbreviation: Incl. = Inclined (PDF) [file pone.0109768.s002.pdf]

Table S2. Significant ANOVA effects involving genotype (Geno) and sex variables from the 1-h locomotor activity test and sensorimotor battery in 4-6 and 12-14 months old *Gnptg*$^{-/-}$ and WT mice.

Test (Age)	Effect	F Statistics
1-h Locomotor Activity		
Ambulations (12-14 mo)	Geno x Time	$F_{(5,75)}$=4.35, p=0.012
	Block 1	$F_{(1,15)}$=5.02, p=0.041
Rearings (12-14 mo)	Geno	$F_{(1,15)}$=4.87, p=0.043
	Block 1	$F_{(1,15)}$=4.59, p=0.049
Sensorimotor Battery		
60º Incl. Screen (4-6 mo)	Geno	$F_{(1,16)}$=18.84, p=0.0005
	Geno x Sex	$F_{(1,16)}$=4.92, p=0.041
90º Incl. Screen (4-6 mos)	Geno	$F_{(1,16)}$=24.65, p=0.0001
90º Incl. Screen (12-14 mos)	Geno	$F_{(1,15)}$=6.14, p=0.026
	Geno x Sex	$F_{(1,15)}$=6.23, p=0.025
Platform (12-14 mos)	Geno	$F_{(1,15)}$=8.62, p=0.010
