# Supplementary material for: Neurologic Abnormalities in Mouse Models of the Lysosomal Storage Disorders Mucolipidosis II and Mucolipidosis III γ
Source: PLoS One. 2014 Oct 14;9(10):e109768. doi: 10.1371/journal.pone.0109768 (PMC4196941; doi:10.1371/journal.pone.0109768)
Supplement: Table S3 — Significant ANOVA effects involving genotype (Geno) and sex variables from the rotarod test in 4–6 and 12–14 months old Gnptg−/− and WT mice. (PDF) [file pone.0109768.s003.pdf]

Table S3. Significant ANOVA effects involving genotype (Geno) and sex variables from the rotarod test in 4-6 and 12-14 months old *Gnptg*$^{-/-}$ and WT mice.

Test (Age)	Effect	F Statistics
Rotarod		
Stationary (4-6 mo)	Geno	$F_{(1,16)}$=6.27, p=0.024
	Geno x Trials	$F_{(2,32)}$=7.85, p=0.003
	Sex x Trials	$F_{(2,32)}$=4.17, p=0.030
	Trial 1	$F_{(1,16)}$=9.38, p=0.007
Constant Speed (4-6 mo)	Geno	$F_{(1,16)}$=10.87, p=0.005
	Session 1, Trial 2	$F_{(1,16)}$=11.71, p=0.004
	Session 2, Trial 2	$F_{(1,16)}$=4.76, p=0.044
	Session 3, Trial 1	$F_{(1,16)}$=6.82, p=0.019
Accelerating (4-6 mos)	Geno	$F_{(1,16)}$=21.61, p=0.0003
	Geno x Session	$F_{(2,32)}$=7.46, p=0.002
	Geno x Sex x Trials	$F_{(1,16)}$=11.01, p=0.004
	Session 1, Trial 2	$F_{(1,16)}$=20.86, p=0.0003
	Session 2, Trial 1	$F_{(1,16)}$=8.04, p=0.012
	Session 2, Trial 2	$F_{(1,16)}$=17.03, p=0.0008
	Session 3, Trial 1	$F_{(1,16)}$=36.73, p<0.00005
	Session 3, Trial 1	$F_{(1,16)}$=8.90, p=0.009
Constant Speed (12-14 mos)	Geno	$F_{(1,15)}$=21.96, p=0.0003
	Session 1, Trial 2	$F_{(1,15)}$=22.42, p=0.0003
	Session 2, Trial 1	$F_{(1,15)}$=5.54, p=0.033
	Session 2, Trial 2	$F_{(1,15)}$=11.65, p=0.004
Accelerating (12-14 mos)	Geno	$F_{(1,15)}$=28.17, p=0.0001
	Geno x Sex x Trials x Sessions	$F_{(2,30)}$=4.71, p=0.017
	Session 1, Trial1	$F_{(1,15)}$=9.78, p=0.007
	Session 1, Trial 2	$F_{(1,15)}$=16.71, p=0.001
	Session 2, Trial 1	$F_{(1,15)}$=14.57, p=0.002
	Session 2, Trial 2	$F_{(1,15)}$=20.64, p=0.0004
	Session 3, Trial 1	$F_{(1,15)}$=12.31, p=0.003
	Session 3, Trial 2	$F_{(1,15)}$=39.41, p<0.00005
